# Supplementary material for: Efficient virus-induced gene silencing in Hibiscus hamabo Sieb. et Zucc. using tobacco rattle virus
Source: PeerJ. 2019 Aug 12;7:e7505. doi: 10.7717/peerj.7505 (PMC6694781; doi:10.7717/peerj.7505)
Supplement: Table S1 [file peerj-07-7505-s001.docx]

**Table S1** The results of sequence identity

| Species | Accession Number | % Identity |
| --- | --- | --- |
| *Gossypium_hirsutum* | NP_001314056.1 | 93.62 |
| *Gossypium_barbadense* | ABN13970.1 | 93.48 |
| *Theobroma_cacao* | EOY06359.1 | 93.20 |
| *Arabidopsis_thaliana* | NP_193291.1 | 87.57 |
| *Populus_tomentosa* | AGT02336.1 | 93.49 |
