# Supplementary material for: Efficient virus-induced gene silencing in Hibiscus hamabo Sieb. et Zucc. using tobacco rattle virus
Source: PeerJ. 2019 Aug 12;7:e7505. doi: 10.7717/peerj.7505 (PMC6694781; doi:10.7717/peerj.7505)
Supplement: Table S2 [file peerj-07-7505-s002.docx]

**Table S2** The average Ct of q-PCR

|  | *18S* | *HhCLA1* |
| --- | --- | --- |
| CK | 15.95507 | 22.24228 |
| CK | 15.95868 | 21.87467 |
| CK | 16.23486 | 21.97121 |
| Mock | 17.703 | 23.58587 |
| Mock | 17.70404 | 23.58587 |
| Mock | 17.705 | 23.3012 |
| 1 | 16.19292 | 24.21782 |
| 1 | 16.40345 | 24.55792 |
| 1 | 16.42291 | 24.19786 |
| 2 | 16.31258 | 23.46068 |
| 2 | 16.46335 | 24.08434 |
| 2 | 16.66926 | 24.21908 |
| 3 | 15.75211 | 23.30857 |
| 3 | 15.80117 | 23.41241 |
| 3 | 16.08517 | 23.32882 |
| 4 | 16.86235 | 24.5888 |
| 4 | 16.95864 | 24.92289 |
| 4 | 16.74121 | 25.33377 |
