# Supplementary figures and images for: Efficient virus-induced gene silencing in Hibiscus hamabo Sieb. et Zucc. using tobacco rattle virus
Source: PeerJ. 2019 Aug 12;7:e7505. doi: 10.7717/peerj.7505 (PMC6694781; doi:10.7717/peerj.7505)

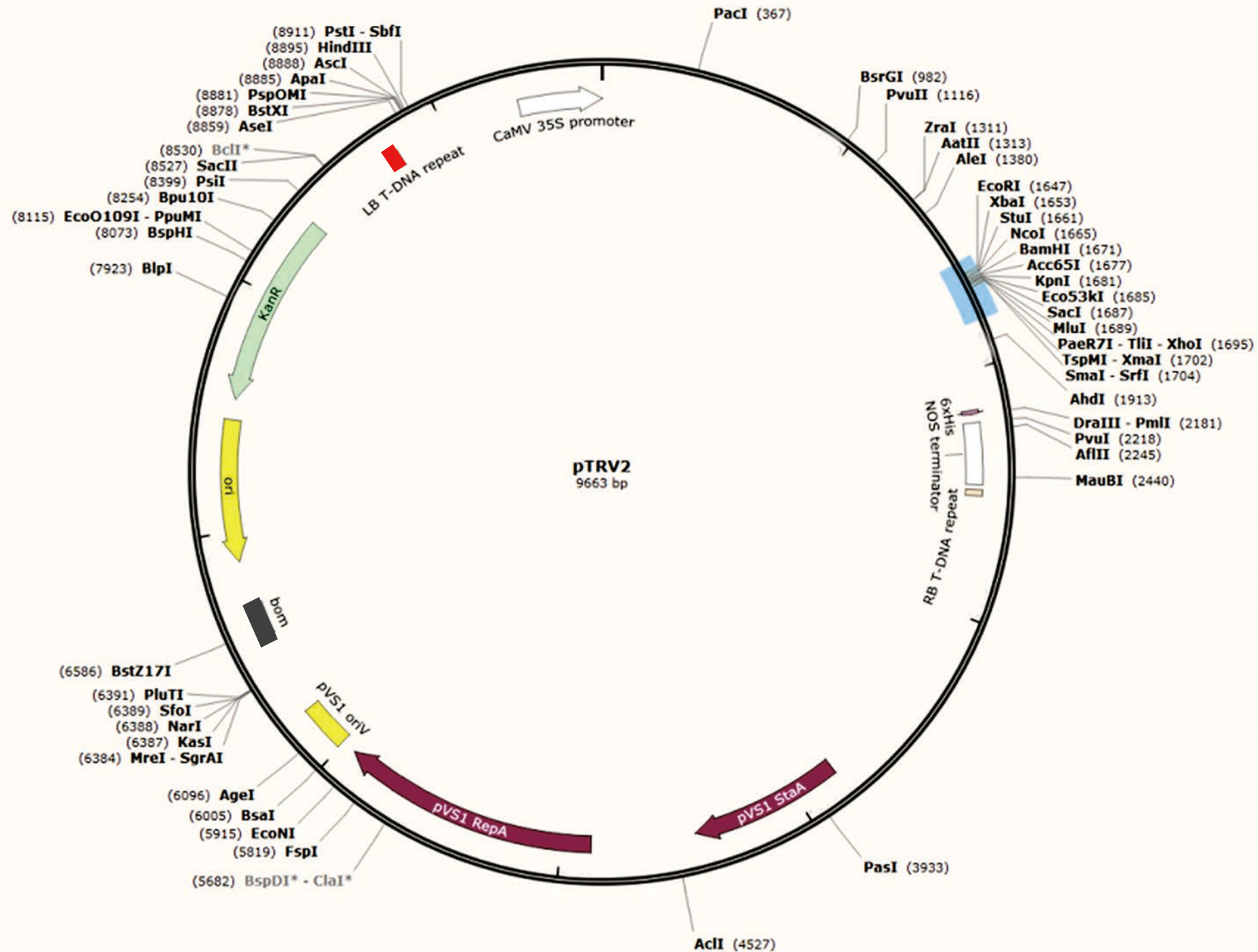

Supplement: Figure S1 [file peerj-07-7505-s003.pdf]

A

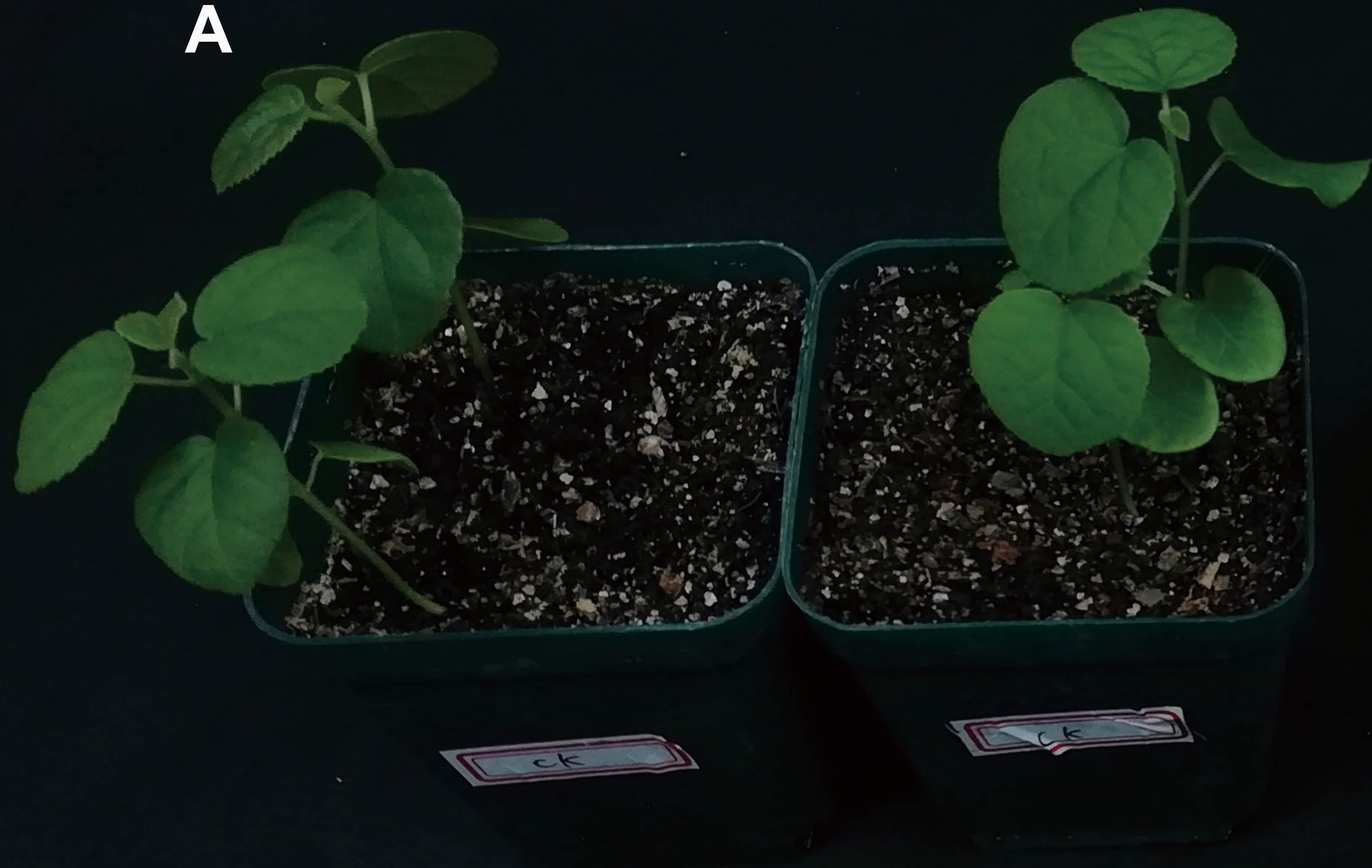

B

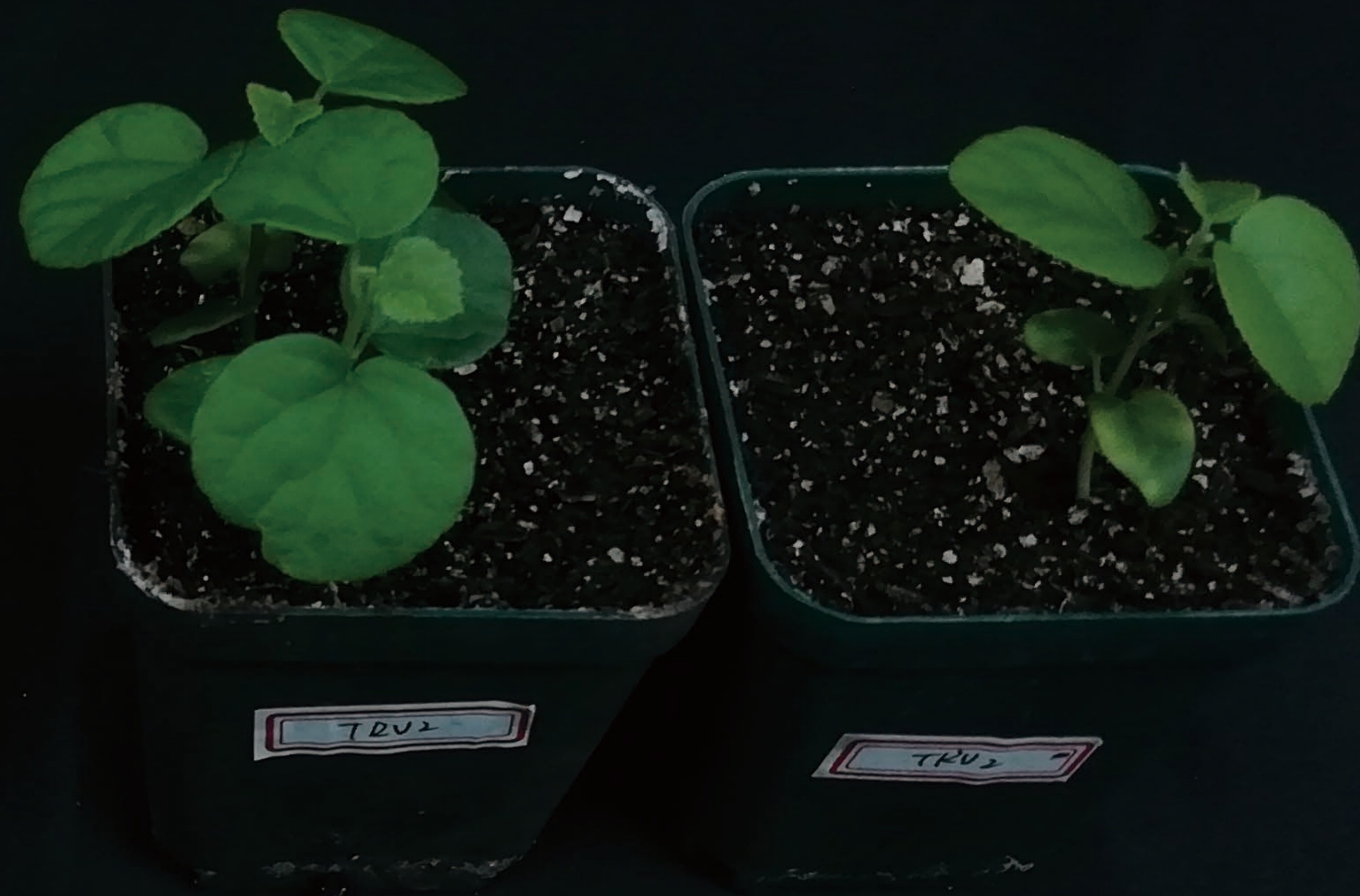

C

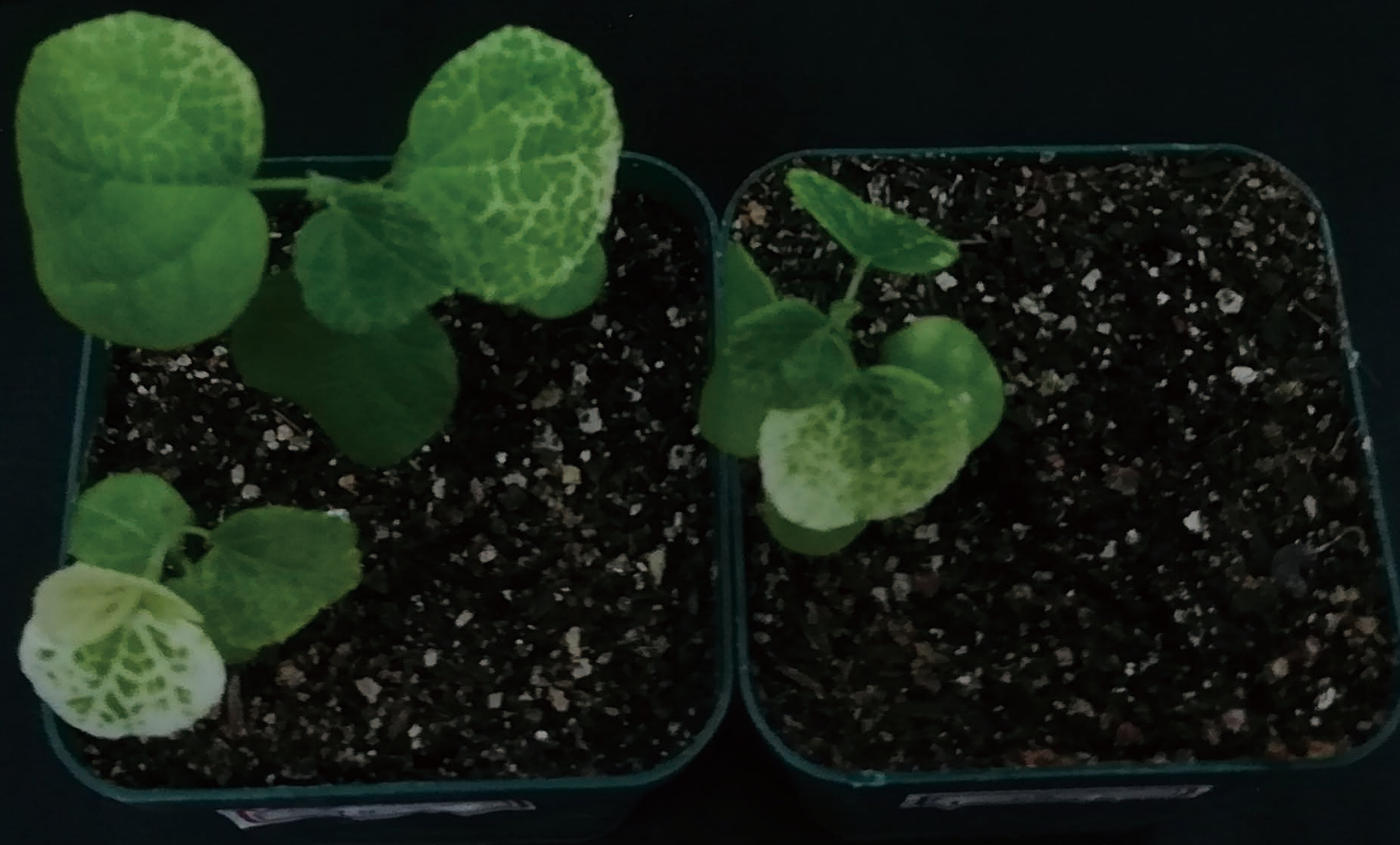

Supplement: Figure S2 — (A) Control plants (CK). (B) Empty vector infiltrated plants (Mock) with the normal phenotype. (C) Newly formed leaves of H. hamabo plants infiltrated with pTRV2-HhCLA1 (CLA1) showing white-streaked leaf symptoms after three weeks. [file peerj-07-7505-s004.pdf]
