# Supplementary material for: Efficient virus-induced gene silencing in Hibiscus hamabo Sieb. et Zucc. using tobacco rattle virus
Source: PeerJ. 2019 Aug 12;7:e7505. doi: 10.7717/peerj.7505 (PMC6694781; doi:10.7717/peerj.7505)
Supplement: Raw data S1 — The sequence of HhCLA, which had been submitted at GenBank, accession no. MK229167. [file peerj-07-7505-s005.docx]

ATGGCTCTTTGTGCATGTTCATTTCCTGTCATTGTTAGCTGCTGTGCTGCTGCTTCAGAACCTCAAAAGTCCTCTCCTTTTGCTTCCCATTTGCTTGCTGGATCAGATCTGGATTTCCACCCATTGCTTAAGCTCAATCAGGTGAAGAAAAGGCCAGGTGGGGTTTATGCATCACTATCAGAAGGAGCTGAATATTACTCCTCAAGACCAGCAACGCCTCTCTTGGACACTATAAACTATCCAATTCATATGAAAAATCTCTCTGTCAAGGAACTGAAACAACTAGCTGATGAACTGCGGTCTGATGTGATTTTCAACGTTTCAAAAACTGGGGGTCACTTGGGGTCAAGCCTTGGCGTGGTTGAACTCACTGTGGCTCTTCATTATGTCTTTAATGCCCCTAGAGACAAGATATTATGGGATGTTGGTCATCAGTCTTACCCTCATAAAATCTTGACTGGGAGAAGAGATAGGATGCATACCATGAGGCAAACTAATGGATTGGCCGGATTCACAAAACGAGCGGAGAGTGAATATGATTGCTTCGGGACTGGTCATAGTTCAACCACAATCTCTGCTGGCTTGGGAATGGCTGTGGGAAGGGATCTGAAAGGTGAAAGGAAACATGTTGTTGCTGTCATAGGCGATGGTGCAATGACTGCGGGACAAGCTTACGAAGCAATGAACAATGCCGGATACCTGGATTCCGATATGATTGTTATTCTTAATGACAATAAACAAGTTTCTCTGCCAACTGCCACTCTTGACGGGCCTATACCGCCTGTTGGAGCTTTGAGCAGTGCTCTCAGTAGGCTGCAATCAAACAGGCCTCTTAGAGAACTGAGAGAGGTTGCAAAGGGAGTTACAAAGCAAATCGGTGGGCCCATGCACGAACTGGCTGCAAAAGTTGATGAGTATGCTCGAGGGATGATAAGTGGTTCCCGTTCAACACTTTTTGAAGAACTTGGACTGTATTATATTGGACCTGTTGATGGCCACAACATCGATGATTTAGTTTCTATTCTCAAAGAGGTTAAGACTACTAAAACAACGGGTCCGGTCTTGATTCATGTTGTCACTGAGAAAGGCCGAGGTTATCCGTATGCAGAGAGAGCTGATGACAAGTACCATGGAGTGGTGAAGTTCGATCCGGCAACTGGAAAGCAATTCAAAGGCAGTTCTGTTACCCAGTCTTACACTACATATTTTGCTGAGGCTTTGATTGCAGAAGCCGAGGCAGACAAAAATATCGTTGCCATCCATGCAGCAATGGGAGGTGGAACTGGATTAAACCTCTTCCTCCGCCGTTTCCCTCAAAGATGTTTCGATGTTGGGATTGCCGAACAACATGCTGTAACCTTTGCTGCAGGGTTGGCGTGTGAAGGCTTGAAACCTTTTTGTGCAATCTACTCATCCTTCATGCAAAGGGCTTATGATCAGGTCGTACACGATGTTGACCTGCAGAAGCTGCCTGTAAGATTTGCTATGGATAGAGCTGGCCTTGTTGGTGCAGATGGTCCGACACATTGTGGGGCTTTCGATGTGACTTTCATGGCATGCCTCCCCAACATGGTCGTAATGGCACCTTCCGATGAGGCTGAGCTTTTTCATATGGTTGCCACAGCTGCAGCCATAGATGACCGTCCTAGCTGTTTCCGTTACCCTAGAGGAAATGGGATTGGAGTTCAGTTGCCGCCAGGGAACAAAGGGGTTCCTCTCGAGATCGGCAAAGGAAGGGTATTGATTGAAGGGGAAAGAGTGGCACTAGTAGGATATGGATCTGCAGTTCAAAGCTGCTTAGCAGCCGCCTCGTTATTGGAATCCCACGGCTTAAAGCTGACTGTCGCGGATGCACGATTCTGTAAACCATTGGATTACTCCCTCCTCCGGGAACTGGCAAAATCACATGAAGTTCTGATCACAGTCGAAGAAGGATCGATCGGGGGCTTCAGTTCTCATGTGGCACAGTTCCTAGCTCTTGATGGTCTTCTTGATGGCAAAGTAAAGTGGAGGCCGGTGGTTCTTCCCGATCGATACATCGATCATGGCTCTCCAGCCGACCAGTTGGCTGAAGCTGGTCTGACACCATCGCACATTGCTGCAACAGTGTTAAACGTGCTTGGAGAAAAAAGAGAGGCTCTTAGGATCATGTCCTCGAGAAAC
